# Foreword to ‘The current status and future perspectives on the management of stage III NSCLC: a focus on unresectable cancer treatment paradigms’

**DOI:** 10.1038/s41416-020-01068-0

**Published:** 2020-12-08

**Authors:** Luis Paz-Ares, on behalf of AstraZeneca UK Limited

**Affiliations:** Hospital Universitario 12 de Octubre, Madrid, Spain

Stage III non-small-cell lung cancer (NSCLC) is a highly complex and heterogeneous disease that presents a number of challenges for achieving optimal treatment outcomes. Stage III disease affects approximately one-third of patients diagnosed with NSCLC each year and prognosis is poor, with a low cure rate. The optimal treatment regimen for patients with NSCLC is most frequently multi-modal, with systemic and local therapies for distant and local disease control, respectively. The exact sequence and modality used is debated and case-specific. The first and most important question in the management of stage III NSCLC, whether the disease is potentially resectable or not, can often be difficult to answer. If the disease is resectable, surgery after preoperative treatment (chemotherapy or chemoradiotherapy (CRT)) is an option, particularly in patients with optimal pulmonary function, mediastinal tumour clearance following upfront treatment and no requirements for pneumonectomy. However, for the remaining patients, or those who have been diagnosed with unresectable stage III NSCLC, platinum-based concurrent chemoradiotherapy (cCRT) is the standard of care versus sequential CRT.^1^

This supplement reviews the clinical evidence for the current European and UK treatment strategies for stage III NSCLC, whilst also providing an overview of the PACIFIC clinical trial (NCT02125461) as the first immunotherapy for unresectable stage III NSCLC whose tumours express PD-L1 on ≥1% of tumour cells following completion of cCRT. This is the first significant treatment advance for these patients in many years.^2^ The supplement concludes with a review of the practical implications, such as prehabilitation and rehabilitation, that could mitigate the potential adverse events of radical therapy.

The first of these articles reviews the key surgical, chemotherapy and radiotherapy trials and guidelines that currently outline radical therapy options for patients with stage III NSCLC. This paper highlights that only one in five of these patients has radical intent multi-modality therapy in the United Kingdom,^3^ and cCRT is not frequently applied. The shared decision-making expertise of the treating multidisciplinary team (MDT) is critical in determining the most appropriate treatment regimen for individual cases, which is cCRT for those with unresectable stage III NSCLC. The scientific rationale for this treatment is based on the results of two large Phase 3 randomised studies and two meta-analyses (predominantly in patients with stage III NSCLC), which demonstrated the superiority of cCRT over sequential chemotherapy and radiotherapy treatment.^1,4–6^ The second article elaborates on how to determine optimal treatable areas for radiotherapy, outlined in the Advisory Committee on Radiation Oncology Practice (ACROP) guidelines,^7^ as well as optimising dose fractionation and chemotherapy regimens to balance the benefits of treatment versus toxicity. In addition, the article discusses the rationale for the benefits that immunotherapies, such as durvalumab (Imfinzi^®^▼; AstraZeneca UK Limited), could provide following cCRT.

In recent years, immunotherapy has gained wide popularity in NSCLC in light of the positive findings from trials of nivolumab (Opdivo^®^; Bristol-Myers Squibb Company, USA) and pembrolizumab (Keytruda^®^; MSD, USA) for the treatment of stage IV metastatic disease. The role of immunotherapy in advanced NSCLC and the rationale for its use following cCRT, in light of the pivotal Phase 3 PACIFIC clinical trial of durvalumab in patients with stage III, locally advanced, unresectable NSCLC whose disease had not advanced following cCRT, is described in this supplement. The PACIFIC study reported significant improvements in the co-primary endpoints of progression-free survival (PFS) and overall survival (OS) following treatment with durvalumab versus placebo in the overall population, as well as in most pre-specified subgroups.^8,9^ Furthermore, the recently reported 3-year overall survival analysis of the PACIFIC study demonstrates long-term clinical benefit with durvalumab following cCRT, further establishing the PACIFIC regimen as the standard of care in this population.^10^ The authors of this supplement conclude that the PACIFIC study, along with ongoing studies of other treatment modalities, are providing significant progress in the treatment of patients with stage III unresectable NSCLC, offering a positive outlook through the advancement of standards of care.

On the basis of the data from the PACIFIC clinical trial,^8^ durvalumab received a positive opinion in Europe by the CHMP on July 26, 2018, and has been recommended for marketing authorisation in Europe. ‘Durvalumab as monotherapy is indicated for the treatment of locally advanced, unresectable NSCLC in adults whose tumours express PD-L1 on ≥1% of tumour cells and whose disease has not progressed following platinum-based chemoradiation therapy’.^9^ Thus, the clinical benefit of durvalumab following cCRT has been formally recognised by the European Medicines Agency (EMA).

However, it is unclear as to whether the set-up of the current standard-of-care procedures and management steps for stage III NSCLC are in place to enable the translation of these results to real-world practice. Increased adoption of cCRT across the United Kingdom will positively impact the proportion of eligible patients with unresectable stage III NSCLC who are able to gain benefit from durvalumab treatment. Patient selection prior to treatment with cCRT will be critical; however, there is currently a lack of comprehensive guidelines for the assessment of patient fitness prior to cCRT. Although often not formally evaluated within the context of clinical trials, there are a number of simple supportive measures (i.e. aggressive symptomatic interventions) that may improve outcomes to cCRT treatment, which is addressed in the final article of this supplement.

In summary, within the heterogeneous treatment landscape for patients with locally advanced, unresectable stage III NSCLC, durvalumab, as a consolidation therapy following two or more cycles of platinum-based cCRT, is emerging as a viable option to treat patients whose tumours express PD-L1 on ≥1% of tumour cells in this setting with curative intent.

## Data Availability

Not applicable.

## Appendix

PRESCRIBING INFORMATION

IMFINZI^®^ ▼(durvalumab) 50 mg/ml solution for infusion

https://medicines.astrazeneca.co.uk/content/dam/multibrand/uk/en/prescribinginformation/imfinzi-pi.pdf
